# Bodies and Bites: a medical school program that teaches anatomy, physiology, and nutrition to elementary school kids

**DOI:** 10.3389/fpubh.2024.1398124

**Published:** 2024-07-09

**Authors:** Kimberly Butterfield, Mary Wesley, Helena Carvalho, Emily Holt, Serkan Toy, Courtney Powell, David Trinkle, Kristofer K. Rau

**Affiliations:** ^1^Carilion Clinic, Roanoke, VA, United States; ^2^Virginia Tech-Carilion School of Medicine, Roanoke, VA, United States

**Keywords:** community engagement, community outreach, service-learning, medical education, STEMM education

## Abstract

Undergraduate medical students who participate in community outreach programs gain a multitude of benefits that impact not only their professional development but also the well-being of the communities they serve. At the Virginia Tech Carilion School of Medicine (VTCSOM), students have the opportunity to volunteer in the “Bodies and Bites” program at the West End Center for Youth, an after-school educational center that serves K-12 children in Roanoke, Virginia. The purpose of Bodies and Bites is to teach elementary school children in 2nd to 5th grade how their bodies work and how to keep them healthy through good nutrition and exercise. All sessions are led by VTCSOM medical students and graduate students from our partnering academic institution, the Fralin Biomedical Research Institute (FBRI). Each week, the children and Health Professions students explore a different topic related to human anatomy and physiology using anatomical models, small group discussions, and hands-on activities. At the end of each session, the participants create a healthy snack related to the day’s topic. The overall goal of the present study was to assess the perception of the Bodies and Bites program from the view of our student volunteers, and the 4th and 5th graders who attend the West End Center. Now in its 6th year, Bodies and Bites continues to be popular as a voluntary program among our Health Professions students, and is well received by the West End Center and the elementary school children they serve. Our students and community mutually benefit from this program, with the former having an opportunity to briefly disengage from the rigors of their studies while gaining valuable skills in science communication and inspiring children to pursue fields in Science, Technology, Engineering, Math, and Medicine (STEMM), and the latter having fun while learning about their bodies and discovering ways to improve their health.

## 1 Introduction and context

A student’s record of consistency and depth of involvement in volunteerism is often among the pillars of successful medical school applications. However, the continuation of such activities has shown to truly benefit these same students as they progress through their Undergraduate Medical Education (UME) (1–5). Since patient contact is often limited for students during their pre-clerkship years, having these experiences is particularly valuable in helping build empathy and compassion, alleviating burn-out, and reminding students of why they wanted to become doctors in the first place (1, 6). One of the most important features of these experiences is establishing that human connection between the Health Professions student and the community members they strive to serve. These community engagements can take a number of different forms, from volunteering time in free clinics, to serving in shelters for individuals experiencing homelessness, to helping out in soup kitchens, and others.

An additional effort to broaden engagement between Health Professions students and their communities has focused on developing programs for children in primary [usually grades K-6 in the United States; e.g., (7, 8)] and secondary education [grades 7–12; e.g., (9–14)]. These programs are typically tailored to the educational level of the children, and often focus on populations that are underserved or underrepresented in medicine. One such opportunity for our local Health Professions students was developed through a unique partnership between the Virginia Cooperative Extension Office,^1^ Virginia Tech-Carilion School of Medicine (VTCSOM),^2^ Radford University-Carilion (RUC),^3^ the Fralin Biomedical Research Institute at Virginia Tech-Carilion (FBRI),^4^ and the West End Center for Youth,^5^ in Roanoke, Virginia. As the on-site location for this partnership, the West End Center for Youth is a non-profit after-school educational program that serves K-12 children who live in one of Roanoke’s most disadvantaged and under-served neighborhoods. The mission of their center is to “strengthen the future of youth by offering holistic programming focused on academics, health and wellness, and cultural arts in a safe and nurturing environment.” The facility contains multiple small classrooms, a large area for dining and working on homework, and a fully equipped kitchen. The West End Center staff, committed to helping children develop the ability to make choices that support their long-term future, rely heavily on visits from external partners and content experts to the center.

To positively impact our community by contributing to the well-being of the children at the West End Center, in 2018, our partnering organizations collaborated on developing the “Bodies and Bites” program. Held during four sessions in the Spring and four in the Fall, this program teaches children in 2nd to 5th grade how their bodies work and how to keep them healthy through good nutrition and exercise. Originally, the Bodies and Bites program served as a primary component of the VTCSOM and RUC Interprofessionalism curriculum, during which Health Professions students (including medical students, student nurses, physician assistants, and allied health providers) were tasked with developing interactive educational programs that promote health to members of our community. The program has since evolved into a purely extracurricular opportunity for medical students at VTCSOM and graduate students at the FBRI to volunteer their time and serve their community in a meaningful way. Program sessions are entirely led by these Health Professions students.

The Bodies and Bites lesson plans focus on different topics for each session, such that the elementary school participants are never exposed to the same lesson plan during each of the 4 years that the Health Professions students work with them. The themes always relate to human anatomy and physiology, as well as nutrition and exercise. Each week, the participants and Health Professions students explore a different theme using anatomical models, small group discussion and hands-on activities. At the end of each session, the children make a healthy snack that is related to the day’s theme.

The overarching goals of the Bodies and Bites program are different for each of the participating groups. For Health Professions students, the program is intended to: (1) Improve and expand the dynamic classroom; (2) Increase student science outreach and engagement with members of the community; and (3) Improve public engagement capabilities to effectively communicate complex science concepts to the public; and (4) Demonstrate positive role modeling and mentorship to promote healthy lifestyles and encourage more students to pursue future careers in STEMM-related fields. For the West End Center children, the program is intended to teach the children to: (1) Have fun while working together as a team; (2) Expand knowledge in science through hands-on activities that demonstrate anatomy and physiology; (3) Identify ways they can keep their bodies healthy and active throughout their lives; and (4) Follow a simple recipe to make a healthy snack.

The relationship that has been established between the Health Professions students, children, West End Center, and other partnering organizations has mutually benefited all invested parties. Evaluation and constructive feedback are regularly requested from everyone involved, including the West End Center staff and administration, which helps ensure that the children, in particular, are given the best possible experience while also benefiting our Health Professions students. We plan to expand and improve the Bodies and Bites program by strengthening collaborative partnerships that are mutually beneficial for academic institutions and the communities they serve.

As the Bodies and Bites program has evolved since it transitioned to a purely voluntary activity outside of the VTCSOM curriculum 3 years ago (e.g., expanded sets of STEMM concepts that are now covered, new hands-on activities, and new recipes), there has not been a regular assessment of the program. Furthermore, this assessment was originally only from the perspective of the Health Professions students. Therefore, the goal of the present study was to assess the Bodies and Bites program from the perspectives of both the Health Professions Student volunteers, as well as 4th and 5th grade children of the West End Center who participated in the program during the Fall 2023 session.

## 2 Key programmatic elements

### 2.1 Overall structure

Prior to their visit to the West End Center, Health Professions students are provided with the lesson plans and have an opportunity to practice the activities that they will be leading. A VTCSOM faculty member designs the lesson plans, purchases and delivers supplies, and remains on-site at the West End Center to provide guidance when necessary; however, the actual sessions are entirely run by the Health Professions students. During the Fall semester, the students work with the combined group of 4th and 5th graders, and during the Spring semester the students work with the combined group of 2nd and 3rd graders. There are typically 8–12 Health Professions students who volunteer per session, and depending on the enrollment at the center, there can be a range of 12–20 participants in each of these groups. This high ratio of Health Professions students to children permits a more conversational and highly engaged interaction among participants.

The program runs for four consecutive Thursday afternoons, each lasting 1.5 h in duration. The children are further split into four smaller groups which are maintained throughout the duration of the program (i.e., the same 3–5 participants per group each week, facilitated by 2–3 Health Professions students). Each group focuses on a different topic each week. All topics are offered concurrently, and groups rotate topics from 1 week to the next. Since there are four total sessions spread across 4 weeks, all of the children have the opportunity to learn about each of the topics.

### 2.2 Lesson plans

Each individual group session begins with a 10-min interactive discussion introducing the day’s topic, why the topic is important in the context of the overall body’s function, and how to maintain health. For the subsequent 40-min, multiple hands-on activities keep the children engaged while demonstrating specific functions. These activities typically include games, crafts, simple experiments, or other high energy activities that get the children moving. For the next 30-min, the Health Professions students assist the children in following a simple recipe to make a healthy snack. Prior to the snack, the children are asked about allergies, since we are doing food prep, we practice good hygiene by having everyone wash hands with soap and warm water, wear gloves, and rinse off any fruits and vegetables required in the recipe. All of the recipes that we use are kid-friendly in simplicity, can be completed within 30 min, and do not require the use of the stove or oven at the facility. The final 10-min are used to answer any final questions and clean up. A full description of the most recent lesson plans that are tailored to 4th and 5th graders are provided as Supplementary material.

Over the 4 years the Health Professions students engage, the West End Center participants progress through four series of topics, each with an age-appropriate, increasing level of challenging concepts and activities that are covered. Set one (tailored to participants in grades 2 and 3) focuses on the senses of sight, hearing, balance, smell, taste, and touch. Set two (grades 2 and 3) focuses on the cardiovascular, digestive, musculoskeletal, and nervous systems. Set three (grades 4 and 5) focuses on cell biology, engineering, genetics, and physiology. Set four (grades 4 and 5) focuses on neuroscience, neurorobotics, neuroprosthetic devices, and artificial intelligence.

### 2.3 Study participants

For the current study, 30 Health Professions student volunteers included VTCSOM medical students from the Classes of 2027 (M1 year of study; *n* = 15), 2026 (M2; *n* = 5), and 2025 (M3; *n* = 1), as well as FBRI graduate students (*n* = 9). Our group worked with all of the 4th and 5th grade children (*n* = 16) who were attending the West End Center in Fall 2023. Not all of these participants were able to attend all four sessions of Bodies and Bites due to occasional absences.

FY2023 total enrollment at VTCSOM is 196 students (49 students per year, four-year curriculum), and for the FBRI is 78 graduate students (~15 students per year; average time to graduation = 5 years), which means for this particular study, 10.7% of VTCSOM students and 11.5% of FBRI students participated in the Fall 2023 Bodies and Bites program. Although it is not a formal component of the curriculum, at VTCSOM there is a service requirement of 30-h for graduation through the VTCSOM Engage service-learning program.^6^ Most Tuesday and Thursday afternoons are free in the curriculum for medical students to study, perform research, or pursue volunteer opportunities. While not required for graduation at the FBRI, volunteer service is highly encouraged for all students. Throughout the year, there are multiple collaborative service opportunities for VTCSOM and FBRI students.

## 3 Assessment and impact metrics

### 3.1 Ethics statement

The requirement of ethical approval was waived by the Institutional Review Board at Virginia Tech for the studies involving humans because the study was considered exempt, as no sensitive or protected data were collected from either the Health Professions students or children. The studies were conducted in accordance with local legislation and institutional requirements.

### 3.2 Health professions student recruitment

Approximately 1 month prior to the first session of Bodies and Bites, an invitation is sent out via email to all VTCSOM medical students and FBRI graduate students. The email contains a description of the program and dates of the sessions. Notices are also posted on bulletin boards with QR codes to sign up online. All students who respond are accepted as volunteers, and no students are turned away. A small proportion of students (approximately 10%) volunteer for multiple sessions during the semester. Approximately 80% of the students will volunteer for multiple semesters. Most of the medical student participation comes from the pre-clerkship years (M1 and M2), although there are typically 2–3 returning students each semester who volunteer during their clerkship years (M3 and M4). Students may add their participation to their CV and receive credit for reporting their volunteer hours (at VTCSOM there is a requirement of 30 h across their 4 our years of UME); however, nothing is formally added to their transcript for participating.

### 3.3 Assessment

For the past several years, Health Professions students have been sent surveys to identify strengths and opportunities for improvement in the Bodies and Bites program. This feedback has helped optimize the activities and recipes that we currently utilize for each of our sessions. Assessment of the program has only recently become much more intentional. During the most recent program (Fall 2023), an anonymous online questionnaire generated in Google Forms was emailed to participating Health Professions students. The survey included open-ended and Likert-style questions after each session to assess their views of the program. The former identified positives and opportunities for improvement:

1. What topic did you lead?
2. What positive(s) did you take away from your experience as a Bodies and Bites facilitator?
3. What challenges did you experience during the Bodies and Bites program related to your group’s lesson plans?
4. What suggestions do you have for ways to improve the Bodies and Bites program?

Likert-style questions were also used to assess overall attitudes toward the program and outreach in general (1–5 scale, where 1 = strongly disagree, 2 = disagree, 3 = neither agree nor disagree; 4 = agree; and 5 = strongly agree):

1. It is important for me to participate in community outreach.
2. After having participated in the Bodies and Bites program, I will definitely participate again in the future.
3. I highly recommend that my classmates participate in the Bodies and Bites program.

We have also started to assess the in-the-moment impact on the children at the West End Center using pre- and post-activity surveys that utilize a colorful 5-face emoticon scale (similar to the Faces Pain Scale used in pediatrics; see Supplementary material). The emoticon scale was then converted into a five-point scale for analysis, ranging from the “very frowny” emoticon = 1, to “very happy” emoticon = 5. To de-identify the children and their assessments, no names were utilized, but rather they were asked to pick a unique color pencil to use for both the pre- and post-activity surveys. The pre-activity survey was given prior to starting the science component, and included the following questions:

1. How do you feel today?
2. Are you interested in this topic?
3. Are you good at science?
4. How much do you like cooking?

The post-activity survey was given prior to cooking, due to Health Professions students sometimes forgetting to distribute these surveys, and the children sometimes having to leave early when their parents or caretakers picked them up. The post-activity survey included the following questions:

1. Was this activity fun?
2. Are you interested in this topic?
3. Are you good at science?
4. Did you learn anything new from the activity?

The only components that were comparable in these surveys were questions #2 (Are you interested in this topic?) and #3 (Are you good at science?). Other questions in the pre-activity survey were intended to (1) assess the general mood of the participants going into the session to determine if their emotional disposition had any effect on answers to subsequent questions (question #1), and (2) qualitatively assess if the participants were interested in cooking in the first place, and whether the broad distribution of these answers changed over the course of the four-week session (question #4). Other questions in the post-activity survey were intended to (1) identify whether the activities were enjoyable or should be modified (question #1), and (2) determine if anything new about the topic was learned from the experience (question #4).

The operations staff at the West End Center is also routinely engaged prior, during, and after each month’s session to determine effectiveness of the program and identify opportunities for improvement.

### 3.4 Statistical measures

The GraphPad online statistical calculator for paired t-tests was used to calculate significance in comparisons between pre- and post-test groups. A two-tailed *p*-value of <0.05 was taken as indicative of significance. Effect size was calculated by dividing the difference between the mean post-test score and the mean pre-test score by the standard deviation of the post-test scores. Data is reported as means (M) and standard deviation (SD) for each group, and when paired t-tests were used, the t test statistic (t), its *p* value (p), and its effect size (d) are listed.

## 4 Results

### 4.1 Assessment from health professions students

Overall, the Bodies and Bites program has consistently received positive feedback from the Health Professions students, West End Center children, and participating organizations. After each session, the Health Professions students were sent links to an anonymous survey. During the previous session, 30 Health Professions students participated in the program, and we received 16 responses to the survey. Two of the 30 students volunteered for two sessions, but were only sent a survey link after their first session, and not their second.

The language used in the open questions by the Health Professions students was assessed for overall conceptual themes. Regarding positives of the program, students expressed enjoyment in interacting with the children (*n* = 14), having the opportunity to teach (*n* = 4), seeing children get excited about STEMM (*n* = 5), seeing children’ attitudes improve during the session (*n* = 2), and flexibility of the lesson plans (*n* = 2). For challenges that were experienced, students identified behavior of some of the children (*n* = 6), time limitations to get through all of the activities (*n* = 4), and difficulty in teaching challenging concepts to children (*n* = 5). Students also suggested ways to improve the program, primarily focusing on ways to improve activities (*n* = 10), recipes (*n* = 2), and classroom arrangement (*n* = 3).

Some sample comments from the session include:

“I really liked how the lesson plan was flexible in terms of instruction and timing so that we can spend more time working with each student or explaining things in detail.”

“I love this experience! The kids are fun, funny, inquisitive, eager, engaged, and wide open! Each time I volunteer, I’m excited about the next time I’m able to volunteer again.”

“One of our kids had previously marked “no interest or experience in science” before the session and marked “full interest” (green happy face) afterwards!”

“It was great to see the kids become so passionate about the topics! One student said she wasn’t very good at science at all, but by the end she said she loved science after all.”

“I gained a lot of respect for the center workers! I was also touched by how excited the kids were by the end, even though several of them started with pretty bad attitudes. It was nice to see their interest grow in such a short time.”

Students were also sent Likert-style questions to assess their overall views of the Bodies and Bites program. During the previous session, most responders selected “Agree” or “Strongly Agree” for each question (Table 1).

**Table 1:** Attitudes of Health Professions students (*n* = 16) toward community outreach and the Bodies and Bites program.

Likert prompt	n	1	2	3	4	5
It is important for me to participate in community outreach.	16	0% (0)	0% (0)	6.25% (1)	12.5% (2)	81.25% (13)
After having participated in the Bodies and Bites program, I will definitely participate again in the future.	16	0% (0)	0% (0)	6.25% (1)	31.25% (5)	62.5% (10)
I highly recommend that my classmates participate in the Bodies and Bites program.	16	0% (0)	0% (0)	0% (0)	25% (4)	75% (12)

All entries are reported as a percentage of the total with number of respondents in parentheses. The assessment was based on a 1–5 scale, where 1 = strongly disagree; 2 = disagree; 3 = neither agree nor disagree; 4 = agree; and 5 = strongly agree.

### 4.2 Assessment from children who attend the West End Center

During the Fall 2023 session, the 4th and 5th grade participants were asked a series of questions to assess their mood and their view of components of the Bodies and Bites program (*n* = 10–13 children per topic and per session; Tables 2–4; see Supplementary material for survey). The averages for each of the survey questions is shown by topic and sequence of session in Tables 2, 3, respectively. For the question concerning “Are you interested in this topic?,” paired *t*-tests did not indicate significant changes in outcome between pre- and post-activity surveys for cell biology (*p* = 0.72, *t* = 0.37, *d* = 0.10), engineering (*p* = 0.28, *t* = 1.15, *d* = 0.44), genetics (*p* = 0.17, *t* = 1.50, *d* = 0.61), or physiology (*p* = 0.64, *t* = 0.48, *d* = 0.25). For the question concerning “Are you good at science?,” paired *t*-tests indicated significant changes in outcome between pre- and post-activity surveys for engineering (*p* = 0.03, *t* = 2.42, *d* = 1.24), but not for cell biology (*p* = 0.64, *t* = 0.49, *d* = 0.19), genetics (*p* = 0.06, *t* = 2.12, *d* = 0.79), or physiology (*p* = 0.47, *t* = 0.74, *d* = 0.29). The percent of children whose ratings between the pre- to post-activity survey decreased, stayed the same, or increased are indicated in Table 4. Sorting data by attitude (“How do you feel today?) did not change significance (data not shown).

**Table 2:** Self-assessment of participants at the West End Center, prior to and following the hands-on activities, organized by topic.

	Pre-activity survey			
Topic	How do you feel today?	Are you interested in this topic?	Are you good at science?	How much do you like cooking?
Cell biology (*n* = 13)	4.2 ± 1.3	3.8 ± 1.1	4.2 ± 1.2	3.9 ± 1.7
Engineering (*n* = 12)	3.7 ± 1.2	3.3 ± 1.7	3.8 ± 1.5	4.3 ± 1.1
Genetics (*n* = 10)	4.3 ± 1.1	3.4 ± 1.6	3.5 ± 1.6	4.0 ± 1.4
Physiology (*n* = 12)	4.5 ± 0.9	4.2 ± 1.3	4.1 ± 1.3	3.8 ± 1.6

	Post-activity survey			
Topic	Was this activity fun?	Are you interested in this topic?	Are you good at science?	Did you learn anything new from the activity?
Cell biology (*n* = 13)	4.5 ± 1.3	4.0 ± 1.5	4.3 ± 0.9	4.1 ± 1.3
Engineering (*n* = 12)	4.4 ± 1.0	3.9 ± 1.5	4.6 ± 0.7	4.0 ± 1.5
Genetics (*n* = 10)	4.8 ± 0.4	4.2 ± 1.3	4.5 ± 1.3	4.2 ± 1.3
Physiology (*n* = 12)	4.6 ± 1.0	4.4 ± 1.0	4.4 ± 1.2	4.4 ± 1.2

Number of participants (n) who participated and completed the pre- and post-test survey is listed next to each topic. Data is reported as mean ± standard deviation on a 5-point scale, from least (1) to most (5) favorable.

**Table 3:** Self-assessment of participants at the West End Center, prior to and following the hands-on activities, organized by sequence of sessions.

	Pre-activity survey			
Session	How do you feel today?	Are you interested in this topic?	Are you good at science?	How much do you like cooking?
1 (*n* = 9)	4.1 ± 1.4	2.4 ± 1.6	4.0 ± 1.6	3.7 ± 1.4
2 (*n* = 11)	4.0 ± 1.3	4.3 ± 1.3	3.5 ± 1.5	3.9 ± 1.5
3 (*n* = 13)	4.2 ± 0.9	4.0 ± 1.2	3.8 ± 1.4	4.4 ± 1.3
4 (*n* = 14)	4.2 ± 1.1	3.7 ± 1.3	4.2 ± 1.2	3.9 ± 1.6

	Post-activity survey			
Session	Was this activity fun?	Are you interested in this topic?	Are you good at science?	Did you learn anything new from the activity?
1 (*n* = 9)	5.0 ± 0.0	4.4 ± 1.1	4.4 ± 1.1	4.8 ± 0.4
2 (*n* = 11)	4.5 ± 1.2	4.7 ± 0.5	4.2 ± 1.3	4.3 ± 1.3
3 (*n* = 13)	4.6 ± 0.9	3.9 ± 1.6	4.6 ± 0.9	4.2 ± 1.4
4 (*n* = 14)	4.2 ± 1.2	3.6 ± 1.5	4.5 ± 0.8	3.6 ± 1.4

Number of participants (n) who participated and completed the pre- and post-test survey is listed next to each topic. Data is reported as mean ± standard deviation on a 5-point scale, from least (1) to most (5) favorable.

**Table 4:** Change between pre- and post-activity self-assessment of participants at the West End Center, for interest in topic and view of being good at science.

	Are you interested in this topic?			Are you good at science?		
	Decrease	Same	Increase	Decrease	Same	Increase
Overall (*n* = 47)	21.3% (10)	38.3% (18)	40.4% (19)	10.6% (5)	53.2% (25)	36.2% (17)
Cell biology (*n* = 13)	23.1% (3)	38.5% (5)	38.5% (5)	15.4% (2)	61.5% (8)	23.1% (3)
Engineering (*n* = 12)	16.7% (2)	30.0% (3)	70.0% (7)	8.3% (1)	41.7% (5)	50.0% (6)
Genetics (*n* = 10)	20.0% (2)	40.0% (4)	40.0% (4)	0.0% (0)	60.0% (6)	40.0% (4)
Physiology (*n* = 12)	25.0% (3)	50.0% (6)	25.0% (3)	16.7% (2)	50.0% (6)	33.3% (4)

All entries are reported as a percentage of the total with number of respondents in parentheses.

### 4.3 Assessment from staff and administration at the West End Center

Although no official surveys are sent to our partners at the West End Center, they regularly provide timely feedback regarding their needs, the organization of the event, and suggestions for improvement. Their staff assists with corrective behavior to the children when necessary, and guides our Health Professions students to improve their interactions with the children. Input from the administration has always been positive and they express their appreciation of the program.

## 5 Discussion

### 5.1 Discussion of results

Based on feedback from the Health Professions students, outreach was viewed as a worthwhile endeavor and in particular, students valued their involvement with the Bodies and Bites program. This is in accordance with other studies that examined the perception of Health Professions students who engaged in community service while in school [e.g., (2, 15, 16)]. Participation in programs that focus specifically on elementary school age children has also been viewed as a positive experience that produces valuable outcomes in these students [e.g., (3, 17, 18)]. The topics that the Health Professions students taught to the West End Center kids focused on the fields of cell biology, engineering, genetics, and physiology.

Children from the West End Center were given a pre-activity survey to assessed their attitudes each day, their interest in the topic, whether they felt they were good at science, and how much they liked cooking. A post-activity survey was also administered to assess whether the children felt the activity was fun, if their interest in the topic had changed, if their self-assessment of being good at science had changed, and if they learned anything new from the activity. When sorting by topic or sequence of session, most of the means of these assessments (63 out of 64) were above 3 on a 5-point scale. The exception was initial interest in the topics during the first session. Although this value increased in the remaining sessions, it did not reach the threshold of significance. Initial interest in the topics of engineering and genetics were also relatively low, whereas cell biology and physiology were viewed more favorably. Statistical analysis was performed on the children’s change in interest in each topic and a self-assessment of whether they were good at science. While the means for these values increased for each topic between the pre- and post-activity survey, only the increase in interest in the topic of engineering was significant. Although perhaps challenging in conceptual content, other higher education centers have similarly tailored outreach programs to the fields covered in this study, pairing their graduate and medical students with local elementary schools [e.g., (19–22)].

### 5.2 Impact

In this study, Health Professions students shared positive views on community engagement, and especially valued their involvement in the Bodies and Bites program. Their feedback has already led to improvements in lesson plans and recipes, making them more engaging and manageable for the children. Children at the West End Center generally had positive attitudes toward the sessions, enjoyed the activities, and maintained or increased their interest in science. Not captured in the surveys was the genuine enthusiasm of the West End Center kids when they saw the Health Professions students arriving at the center each day. They frequently expressed their excitement for “Bodies and Bites” day each week.

Overall, there are multiple benefits to all participants in programs like Bodies and Bites. Through interactions with young children in small groups, professional students hone the necessary skills to communicate complex science concepts to the public and promote healthy lifestyles (23–25). Children in elementary school similarly benefit from such experiences. They are naturally curious about their bodies and can easily relate to activities involving anatomy and physiology, so beginning to teach these topics at an early age fosters numerous positive outcomes, including improved perception to learn, reduced propagation of health misinformation, better awareness of the dangerous effects of drugs and alcohol, increased interest in STEMM-related fields, and others (26, 27). Furthermore, studies have also demonstrated the positive impact of programs in which Health Professions students teach elementary school children about nutrition, culinary skills, and hygiene (28). Such programs not only teach children about ways to maintain a healthy lifestyle, but also increase student confidence in providing nutrition and obesity counseling for their future patients.

### 5.3 Challenges, limitations, and future directions

Challenges associated with the program include keeping energetic children focused and dealing with tiredness and hunger. This was the impetus for developing lesson plan activities that piqued curiosity and involved movement. During cooking, limited supplies at times made taking turns difficult. Nonetheless, everyone who participated seemed to enjoy the experience, and students learned that working with the kids required creativity, adaptability, and communication, which are all useful skills for future careers in medicine and research.

Although the Bodies and Bites program continues to be successful, other significant challenges exist. As this program has transitioned from being embedded within the curriculum to voluntary, one of the primary challenges is establishing a reliable funding mechanism to support the costs of materials and food, which is approximately $500 USD per semester. Currently, funding is acquired through small internal grants at Virginia Tech, as well as support from VTCSOM. Another challenge relates to recruiting student volunteers in sufficient numbers to staff each of the stations. With in-class requirements and out-of-class studying, demands on the time of Health Professions students are often quite high. Furthermore, to maximize effectiveness, long-lasting impact, and mutually benefit all invested partners, it is vital that outreach programs such as Bodies and Bites are not only designed in partnership with community stakeholders, but that there are additional opportunities to reflect on these experiences (29). A service-learning reflection is not currently associated with the Bodies and Bites program.

It is acknowledged that Health Professions students have numerous opportunities for community outreach in the local area. Some students participate in Bodies and Bites a single time whereas others regularly volunteer one or more times per session. Also, the children of the West End Center undoubtedly have other exposures to similar topics in STEMM and activities that are used in the Bodies and Bites program. The surveys used in this study were designed to gauge in-the-moment satisfaction with the program for the Health Professions students and the children. This is not sufficient to assess overall attitudes toward community service or science, knowledge gained, or impact. Therefore, any direct effects of the program are difficult to determine at this juncture. A more longitudinal, repeated measures design could be used to better track how knowledge and attitudes evolve over time. We are currently not permitted to track individual kids at the West End Center as they progress from grade to grade.

As it pertains to this study, the biggest limitation is the very small sample size. While we worked with all of the 4th and 5th graders who currently attend the West End Center (*n* = 16), not all of the kids were able to complete all four sessions due to absences or being picked up early by their guardians. The structure of the Bodies and Bites program is such that the 2nd-5th grade kids that we work with will have different experiences in terms of topics, activities, and recipes in each grade (i.e., the Fall 2023 program will not be run again until Fall 2025). Regarding the participation of Health Professions students, we similarly had a small sample size, although considering the relatively small population of VTCSOM medical students and FBRI graduate students, as well as other opportunities for community engagement, participation by approximately 10% of students was judged to be successful. Due to limitations of space at the West End Center, it would also be challenging to accommodate more students for each session.

Future directions of the Bodies and Bites program will aim to expand upon the content and engage with children in the middle school age. We are also in the process of developing relationships with other after-school programs in the area. Although we have begun to assess the program more robustly, we are also curious as to whether the learning extends beyond our time in the West End Center. For that reason, we intend to survey parents regarding what the kids discuss about their experience in the program at home.

## Data availability statement

The raw data supporting the conclusions of this article will be made available by the authors, without undue reservation.

## Ethics statement

The studies involving humans were approved by the Virginia Tech Institutional Review Board. The studies were conducted in accordance with the local legislation and institutional requirements. Written informed consent for participation in this study was provided by the participants’ legal guardians/next of kin. Written informed consent was obtained from the individual(s) for the publication of any potentially identifiable images or data included in this article.

## Author contributions

KB: Conceptualization, Methodology, Writing – review & editing. MW: Project administration, Supervision, Writing – review & editing. HC: Project administration, Supervision, Writing – review & editing. EH: Project administration, Supervision, Writing – review & editing. ST: Formal analysis, Writing – review & editing. CP: Project administration, Resources, Supervision, Writing – review & editing. DT: Conceptualization, Writing – review & editing. KR: Conceptualization, Data curation, Formal analysis, Funding acquisition, Investigation, Methodology, Project administration, Resources, Supervision, Writing – original draft.

## Footnotes

^1^ https://www.virginia.gov/agencies/virginia-cooperative-extension/

^2^ https://medicine.vtc.vt.edu/

^3^ https://www.radford.edu/content/ruc/home.html

^4^ https://fbri.vtc.vt.edu/

^5^ https://www.westendcenter.org/

^6^ https://medicine.vtc.vt.edu/community/vtc-engage.html
