# Supplementary material for: Bodies and Bites: a medical school program that teaches anatomy, physiology, and nutrition to elementary school kids
Source: Front Public Health. 2024 Jul 9;12:1398124. doi: 10.3389/fpubh.2024.1398124 (PMC11263331; doi:10.3389/fpubh.2024.1398124)
Supplement: Supplementary file 1 [file Data_Sheet_1.docx]

**Supplementary Material**

**S1** Pre-test and post-test questionnaire for the children.

**
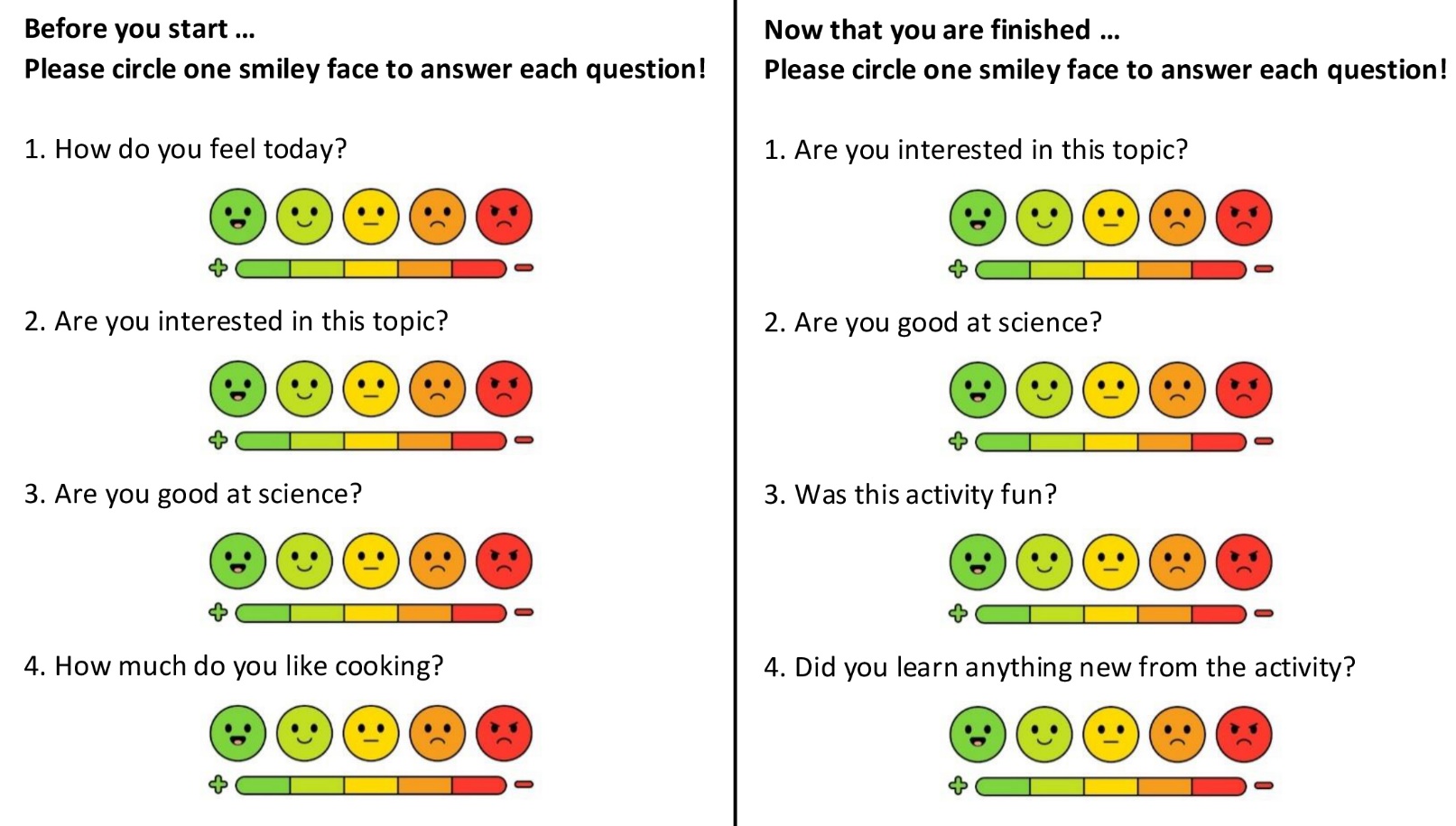
**

**S2** Lesson plan for the Bodies & Bites session on cell biology.

**Bodies & Bites CELL BIOLOGY: Overview**

**Have students fill out the “Before you start…” survey.**

**First 10 minutes** – Introductions and general discussion on the field of cell biology.

- Ask students, “*What do you know about cell biology?*” Explain that cell biology is the study of cell structure and function. The cell is the smallest unit with the basic properties of life. Some tiny organisms, such as bacteria and yeast, consist of only one cell. Large plants and animals have many billions of cells. Human beings are made up of more than 75 trillion cells.
- Using plastic models and images, explain some of the parts and functions, and compare/contrast the similarities/differences between the plant and animal cells (note: images and descriptions of the cell parts are provided as separate documents, which are not included here).
- Introduce the activities for the lesson and review the instructions.

**Next 40 minutes** – Rotate through the following interactive demonstrations:

**A. Build a cell** (15 minutes): Make a Shrinky Dinks^©^ cell

Materials Needed:

- Animal and plant model cells (purchased from <https://biologyproducts.com/>)
- Shrinky Dinks^©^ paper (purchased from <https://www.michaels.com/>)
- felt-tipped pens
- baking pan
- parchment paper
- oven mitt

**B. Examine cells under the microscope** (25 minutes):

Materials Needed:

- Paper towels
- Neoprene gloves
- Microscope slides and cover slips
- Toothpicks or cotton swabs
- Dropper or pipette
- Paper plates
- Light microscope (purchased from <https://amscope.com/>)
- Prepared slides (purchased from <https://amscope.com/>)

**Have students fill out the “Now that you are finished…” survey.**

**Next 30 minutes** – Make a healthy snack

**Last 10 minutes** – Clean up and answer final questions

**Bodies & Bites CELL BIOLOGY: Activities**

**A. Build a cell** (15 minutes):

1. Have students choose which type of cell they would like to make (plant or animal).

2. Each student should tape the image to the back of their Shrinky Dink^©^ sheet.

3. Color the Shrinky Dink^©^ sheet with alcohol-based permanent markers.

4. At some point while the students are coloring, you should pre-heat the oven (set at 325°F).

5. If the students want just the cell to be shrunk, they should outline the cell in marker, and then cut it out with scissors (the whole Shrinky Dink^©^ sheet can be shrunk too as an alternative).

6. Use a baking parchment paper to line the baking pan.

7. Place the Shrinky Dink^©^ sheets colored side up on the lined tray.

8. Important step! If this is a convection oven, place a second piece of baking parchment paper on top of the Shrinky Dink^©^ sheets, and then a second baking pan on top of that. Otherwise, the Shrinky Dinks^©^ just blow around inside the oven!

9. Put the trays into the pre-heated oven.

10. Bake for 3 minutes. After the pieces lie flat, allow an additional 30 seconds of baking time to complete the process, then remove from oven (with an oven mitt!!!!).

11. Remove the baking parchment paper from the baking tray to allow to cool for a few minutes. If the pieces did not flatten out completely you can lightly press the pieces flat with a folded paper for about 15 seconds until they have completely cooled.

12. Turn off the oven!

13. The kids can take their Shrinky Dinks^©^ cells with them.

**B. Examine cells under the microscope** (25 minutes):

*It is a good idea to have one adult paired with a microscope. Kids can rotate viewing slides.*

1. View prepared slides of various tissues and cells under the microscope.

2. View human cheek cells under the microscope.

Collecting the Sample

Prepare the slide: Place a clean microscope slide on a paper plate.

Collect cheek cells: Have the student gently rub the inside of their cheek with a toothpick or sterile cotton swab. It’s important to be gentle to avoid irritation.

Transfer cells to the slide: Rub the toothpick/cotton swab across the center of the slide in a to transfer the cheek cells onto the slide. *Note: the area of transfer can only be as big as the coverslip.*

Staining the Cells

Apply the stain *(Best to have the adult do this step, since stain can get on clothes and hands)*: Use a dropper or pipette to place a single drop of stain on the slide where the cheek cells were applied. This stain will help to make the cells and their nuclei visible under the microscope.

Wait: Let the stain sit on the slide for about 1 minute. This gives the stain enough time to penetrate the cells.

Rinse the slide: Gently rinse the slide with a few drops of water to remove excess stain. This step should be done carefully to avoid washing away the cheek cells.

Remove excess water: Gently dab the edge of the slide with paper towels to remove any remaining water.

Preparing the Slide for Viewing

Cover the sample (*Probably best to have the adult do this step*): Carefully place a cover slip over the stained area to protect the sample and to flatten it for easier viewing. Try to avoid creating air bubbles.

Viewing the Sample

Set up the microscope: Place the slide on the microscope stage and secure it with the stage clips.

Start with low power: Begin viewing the sample under the lowest magnification to find the stained cells.

Increase magnification: Once you’ve located the cells, switch to a higher magnification for a closer look. Adjust the focus carefully to see the cell details, particularly the stained nuclei.

**Bodies & Bites CELL BIOLOGY: Recipe**

**Rice Cake Cell Model**

**Next 30 minutes** – Ask about any allergies. Since we are doing food prep, practice good hygiene: have everyone wash hands with soap and warm water, and rinse off any fruits and vegetables required in the recipe. Make a healthy snack (**Rice Cake Cell Model**) using foods that are high in healthy nutrients. Talk to students about other foods that are healthy for their bodies and that they like to eat.

**Materials**

- cutting board
- knife to slice fruit
- spatula for spreading peanut butter or cream cheese
- paper plates
- paper towels

**Ingredients**

- 1 rice cake per person
- shredded coconut
- bananas
- strawberries
- raisins
- creamy peanut butter (or sun butter)
- granola

**Directions**

1. Spread peanut butter (or sun butter if there are allergies) on a rice cake (if desired).

2. Add other ingredients as you desire to represent different parts of a cell.

3. Enjoy your treat!

**Last 10 minutes** – Clean up and answer final questions

**S3** Lesson plan for the Bodies & Bites session on engineering.

**Bodies & Bites ENGINEERING: Overview**

**Have students fill out the “Before you start…” survey.**

**First 10 minutes** – Introductions and general discussion on the field of engineering and how it relates to the human body.

- *Ask students, “What do you know about engineering?”* Explain that engineering is the branch of science and technology concerned with the design, building, and use of engines, machines, and structures to make things like bridges, buildings, circuits, etc.
- Ask the students if they can think of any parts of the body where engineering might be important. Use available diagrams and models. Some possibilities might include:
  - Having parts that are made to be strong (e.g., bones), others to be flexible (e.g., muscles, tendons, ligaments, cartilage), and others that combine strength and flexibility (e.g., the vertebrae, intervertebral disks, joints, soft tissues, nerves and spinal cord that form the spine).
  - There are multiple examples of simple machines in the human body. A “simple machine” is a mechanical device that changes the direction or magnitude of a force. In general, they can be defined as the simplest mechanisms that use mechanical advantage (also called leverage) to multiply force. Usually, the term refers to the six classical simple machines: Lever, Wheel and axle, Pulley, Inclined plane, Wedge, Screw (note: images of each of these machines are shown to the children, and were acquired from online image searches).
- Introduce the activities for the lesson and review the instructions.

**Next 40 minutes** – Rotate through the following interactive demonstrations:

**A. Build simple machines out of LEGO that model parts of the body** (20 minutes):

Materials Needed:

• LEGO bricks

**B. Build a spinal bridge** (20 minutes):

Materials Needed:

• popsicle sticks • tape

• pipe cleaners • cups

• clothespins • weights (e.g., pennies)

• straws

**Have students fill out the “Now that you are finished…” survey.**

**Next 30 minutes** – Make a healthy snack

**Last 10 minutes** – Clean up and answer final questions

**Bodies & Bites ENGINEERING: Activities**

**A. Build simple machines out of LEGO that model parts of the body** (20 minutes):

Simple machines make work easier and help move heavy loads. As complex machines, our bodies do an amazing job of coordinating these simple machines with other systems; yet sometimes things go wrong. Misdirected forces may make work less efficient or cause strain and injury. Reminders to “lift with the legs” or “stand up straight” are actually ways to keep our body machines in balance. Understanding the simple machines at work in our bodies is important. **Use LEGO to make a simple machine.**

**Examples of Simple Machines in the Human Body**

- Levers: bars that are free to pivot or move about a fixed point (the fulcrum) when a force is applied. They help us to lift things more easily by pivoting about a fulcrum. Our bones, joints and muscles act as levers, with elbows and the balls of our feet as fulcrums.
- Wheels and axles: used primarily to enhance range of motion and speed of movement. Ball-and-socket joints in our shoulders and hips aren’t true wheels and axles, but serve a similar function. The humerus acts as an axle and rotator cuff the wheel because when you rotate it slightly, the humerus moves with it. Simple machines like this help us throw things further distances while using less force.
- Pulleys: change the direction of an applied force. Our tendons and kneecap form a pulley system that redirects force, keeping our bones from crunching together as we lift and bend the lower leg.
- Inclined planes: are a flat surface that is slanted. It is typically used to help move/lift heavy loads. Our feet have an inclined plane on the surface and underneath them. Our feet can hold up a lot of weight, and the inclined plane helps to keep us balanced and sturdy.
- Wedges: Wedges work by putting a narrow end into something we want to come apart, and applying force to the wider end. Our teeth are an example of a wedge found in our body. When we eat, we put our bottom teeth into the middle of the food and break it apart with our top teeth.
- Screws: A screw is like a twisted inclined plane. If you experience a bad break of a bone, especially on your ankle, the doctor might operate on you and give you a syndesmotic screw. These screws help to realign the bones and get you back on your feet.

**B. Build a spinal bridge** (20 minutes):

What makes your backbone strong? A backbone is like a bridge, because it is long and can support a lot of weight. It is also made in such a way that it is very strong (show pictures of what bone looks like under the microscope; also show the vertebrae). Students will make a bridge of their own out of the materials provided. They must use at least three materials. At the end we will try to figure out which bridge is the strongest by placing a cup on the bridge and slowly adding weight.

**Bodies & Bites ENGINEERING: Recipe**

**Hummus Veggie Wrap**

**Next 30 minutes** – Ask about any allergies. Since we are doing food prep, practice good hygiene: have everyone wash hands with soap and warm water, and rinse off any vegetables required in the recipe. Make a healthy snack (Hummus Veggie Wrap) using foods that are high in nutrients. Talk to students about other foods that are healthy for their bodies and that they like to eat.

**Materials**

- cutting board • paper plates
- knife for cutting vegetables • paper towels
- spatula or utensil for spreading hummus • eating utensils

**Ingredients**

- flour tortilla wraps • carrot (julienned)
- hummus • cucumber (sliced)
- feta cheese • bell pepper (sliced)
- 1 cup plain Greek yogurt • 2 tablespoons [honey](https://greatist.com/eat/difference-between-natural-sweeteners)

**Directions**

1. Make a dressing by combining the Greek yogurt and honey in a bowl.
2. On each tortilla wrap, spread a few tablespoons of hummus.
3. Layer the sliced cucumber, bell pepper, and carrot. Add feta cheese if desired.
4. Top with a moderate amount of dressing (everyone share!).
5. Fold the sides of the tortilla inward over the fillings. You want to tuck these in so nothing falls out.
6. Enjoy your treat, and when finished please help clean-up!

**Last 10 minutes** – Clean up and answer final questions

**S4** Lesson plan for the Bodies & Bites session on genetics.

**Bodies & Bites GENETICS: Overview**

**Have students fill out the “Before you start…” survey.**

**First 10 minutes** – Introductions and general discussion on the field of genetics.

- Ask students, *“What do you know about genetics?”* Explain that genetics is the study of how genes and how traits are passed down from one generation to the next. Our genes carry information that affects our health, our appearance, and even our personality!
- Ask students, *“Do any of you know what DNA is?”* Explain that DNA is the genetic information inside the body's cells that helps make people who they are. It's the instructions for how to make the body, like the code to a video game or blueprints for a house. If you used a very strong microscope, you would see that DNA looks like a twisting ladder. You can use the diagrams provided in this lesson plan to give the students an idea of the double-helix shape and the arrangement of nitrogenous bases (cytosine and guanine, and thymine and adenine).
- Introduce the activities for the lesson and review the instructions.

**Next 40 minutes** – Rotate through the following interactive demonstrations:

**A. Extract DNA** (20 minutes): Extract DNA from strawberries.

Materials Needed:

• strawberries • coffee filter

• Ziplock bags • dish detergent

• coffee stirrer • salt

• plastic cups • rubbing alcohol

• pitcher • water

**B. Build a DNA model** (20 minutes): Make a DNA keychain using a key ring, pipe cleaners, and beads.

Materials Needed:

• key rings • beads

• pipe cleaners • scissors

**Have students fill out the “Now that you are finished…” survey.**

**Next 30 minutes** – Make a healthy snack

**Last 10 minutes** – Clean up and answer final questions

**Bodies & Bites GENETICS: Activities**

**A. Extract DNA** (15 minutes): Utilize the directions provided by the National Human Genome Research Institute to extract DNA from strawberries (<https://www.genome.gov/about-genomics/teaching-tools/strawberry-dna-extraction>).

**B. Build a DNA model**


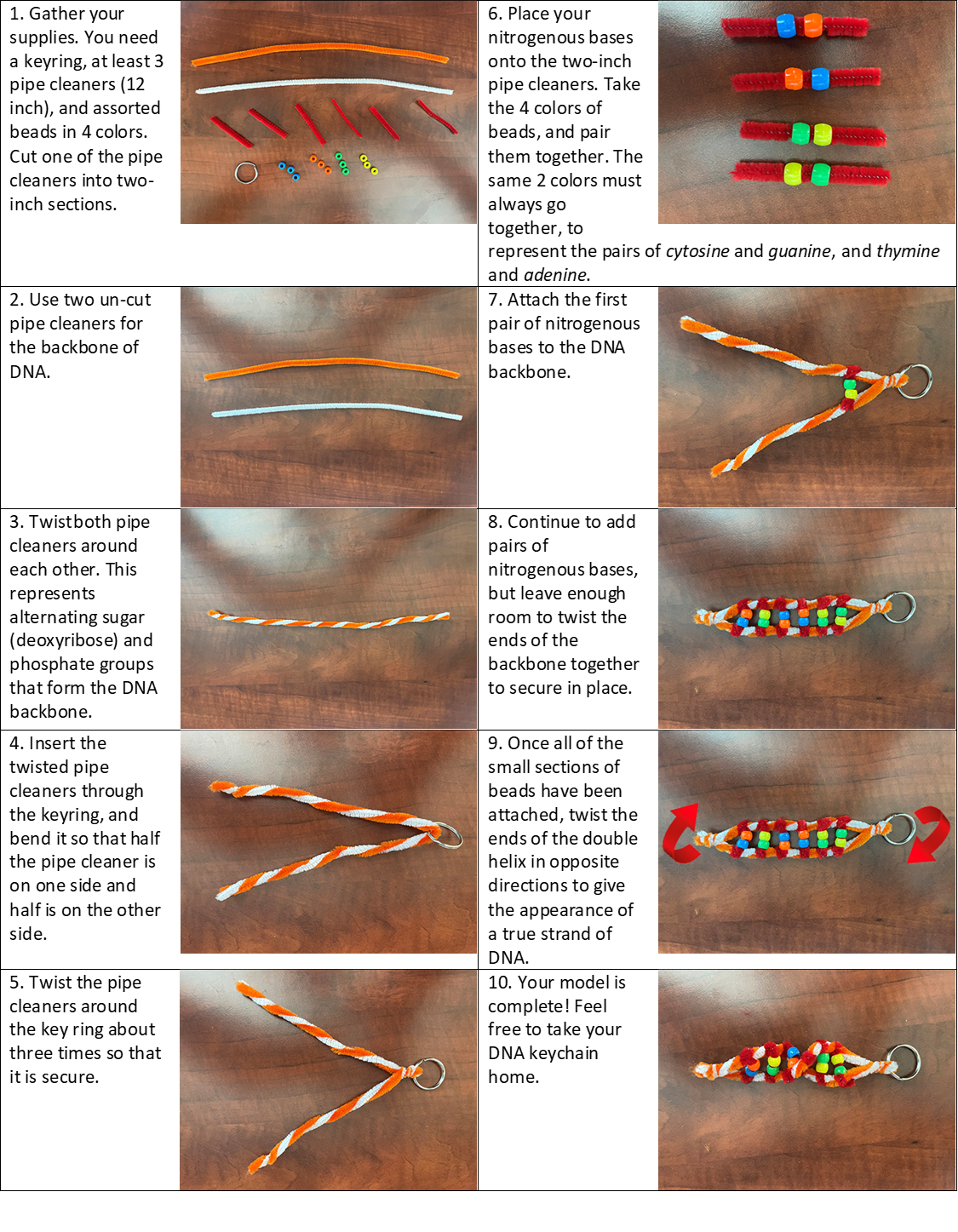


**Bodies & Bites GENETICS: Recipe**

**No-Bake Energy Bites**

**Next 30 minutes** – Ask about any allergies. Since we are doing food prep, practice good hygiene: have everyone wash hands with soap and warm water, wear gloves, and rinse off any fruits and vegetables required in the recipe. Make a healthy snack (**No-Bake Energy Bites**) using foods that are high in nutrients. Talk to students about other foods that are healthy for their bodies and that they like to eat.

**Materials**

• mixing bowl

• mixing spoon

• 1/3 cup, 1/2 cup, and 1 cup

• 1 tablespoon

• 1 teaspoon

• paper plates

• paper towels

**Ingredients**

• 1 cup old-fashioned oats • 1/2 cup vegan chocolate chips

• 2/3 cup toasted shredded coconut • 1/3 cup honey

• 1/2 cup creamy peanut butter • 1 tablespoon chia seeds

• 1/2 cup ground flaxseed • 1 teaspoon vanilla extract

**Directions**

1. Stir all ingredients together in a large mixing bowl until thoroughly combined.

2. Roll into mixture into 1-inch balls.

3. Enjoy your treat, and when finished please help clean-up!

**Last 10 minutes** – Clean up and answer final questions

**S5** Lesson plan for the Bodies & Bites session on physiology.

**Bodies & Bites PHYSIOLOGY: Overview**

**Have students fill out the “Before you start…” survey.**

**First 10 minutes** – Introductions and general discussion on the field of physiology.

- *Ask students, “What do you know about physiology?”* Explain that physiology is the study of how the human body works. It describes the chemistry and physics behind basic body functions, from how molecules behave in cells to how systems of organs work together. It helps us understand what happens in a healthy body in everyday life and what goes wrong when someone gets sick.
- *Ask students “What is the cardiovascular system and what does it do?”* The cardiovascular system is your heart and blood vessels. The cardiovascular system supplies your body's organs with oxygen and nutrients so your organs can do their jobs. Your blood vessels also carry carbon dioxide and other wastes away for disposal. Use diagrams and anatomical models to illustrate the cardiovascular system.
- *Ask students “What is the respiratory system and what does it do?”* The respiratory system includes your lungs and other parts of your body involved in breathing. The respiratory system works closely with your cardiovascular to supply your blood vessels with oxygen, and remove carbon dioxide. Use diagrams and anatomical models to illustrate the respiratory system.
- Introduce the activities for the lesson and review the instructions.

**Next 40 minutes** – Rotate through the following interactive demonstrations:

**A. Listen to your heartbeat (10 minutes):** Use stethoscopes to listen to your heartbeat.

Materials needed: • stethoscopes

**B. Heart and breathing rate experiment (30 minutes):** Today we will be scientists and do an experiment to test how our cardiovascular and respiratory systems work before and after exercise.

Materials Needed:

• toothpicks • dice • stickers

• Play Dough • record sheets • pencils

**Have students fill out the “Now that you are finished…” survey.**

**Next 30 minutes** – Make a healthy snack

**Last 10 minutes** – Clean up and answer final questions

**Bodies & Bites PHYSIOLOGY: Activities**

**A. Listen to your heartbeat** (10 minutes): Demonstrate how to use stethoscopes to listen to your heartbeat.

**B. Heart and breathing rate experiment** (30 minutes): Each kid is provided an Experiment Record Sheet to keep track of their heart rate and breathing rate data.

**Experiment Record Sheet**

|  | **Resting** | **After Exercise** |
| --- | --- | --- |
| **Heart rate**  (beats per minute) |  |  |
| **Breathing rate**  (breaths per minute) |  |  |

**Heart Rate Experiment:**

GETTING TO THE HEART OF THE MATTER: Each time the human heart beats, blood is pumped through the arteries to the lungs and rest of the body. As blood is forced through the arteries during a heartbeat, the artery stretches and bulges slightly. This brief bulge is called a **pulse**, and can be felt in arteries on your wrist or neck. You can measure your **heart rate** by counting the number of pulses in a minute (or by counting for 30 seconds and multiplying by two). Your heart rate can change depending on physical condition, diet, and current activity. It can also change with external stimuli, such as being frightened unexpectedly or being placed in a calming room with soothing, relaxing music.

Ask the students the following questions:

*What are two activities in which you participate that might increase heart rate?*

*What are two activities in which you participate might decrease heart rate?*

1. Have the students each make a pulse rate monitor out a toothpick, Play Dough, and sticker (the sticker is optional … it just helps make the movement more obvious). Show the students where to place the Play Dough so that they can see the pulse on their wrist (refer to picture to the right).

2. Everyone should determine their “resting heart rate” using the pulse rate monitor. Count the number of pulses over 30 seconds. Multiply that number by two for the number of *beats per minute*. Use the **Experiment Record Sheet** (see table above) to record this number.

3. Next everyone should roll a dice to determine which exercise they are supposed to do. Depending on what they roll they need to do one of the following activities for 60 seconds straight (no breaks!):

1 – Jumping jacks

2 – Sit ups or crunches

3 – Push ups

4 – Jog in place

5 – Dance

6 – High knees or butt kicks (your own butt, not your friend’s butt)

4. After doing the exercise, everyone should immediately determine their “active heart rate” using the pulse rate monitor. Count the number of pulses over 30 seconds. Multiply that number by two for the number of *beats per minute*. Use the Experiment Record Sheet to record this number.

**Bodies & Bites PHYSIOLOGY: Activities (continued)**

**Breathing Rate Experiment:**

A BREATH OF FRESH AIR: Respiration is also closely linked with your heart. As you inhale and bring air into your lungs, your **pulmonary artery** brings blood in need of oxygen to the lungs. Through tiny blood vessels called **capillaries**, blood is brought into contact with the alveoli (air sacs) of the lungs. Oxygen diffuses out of the alveoli and into the blood, while carbon dioxide diffuses out of the blood and into the **alveoli**. As you exhale, you get rid of this carbon dioxide. Oxygenated blood is carried back to the heart through the **pulmonary vein**, and is then pumped by the heart to the rest of the body. You can calculate your **breathing rate** by counting the number of breaths you take each minute. Your respiration rate will also change depending on external stimuli, current activity and your level of physical fitness.

Ask the students the following questions:

*What are two activities that might increase your rate of respiration?*

*What are two activities that might decrease your rate of respiration?*

1. Everyone should determine their “resting breathing rate”. Count the number of breaths over 30 seconds. Multiply that number by two for the number of *breaths per minute*. Use the **Experiment Record Sheet** to record this number.

2. Next everyone should roll a dice to determine which exercise they are supposed to do. Depending on what they roll they need to do one of the following activities for 60 seconds straight (no breaks!):

1 – Jumping jacks

2 – Sit ups or crunches

3 – Push ups

4 – Jog in place

5 – Dance

6 – High knees or butt kicks (your own butt, not your friend’s butt)

3. After doing the exercise, everyone should immediately determine their “active breath rate”. Count the number of breaths over 30 seconds. Multiply that number by two for the number of *breaths per minute*. Use the Experiment Record Sheet to record this number.

**Bodies & Bites PHYSIOLOGY: Recipe**

**Banana Split Pudding Cups**

**Next 30 minutes** – Ask about any allergies. Since we are doing food prep, practice good hygiene: have everyone wash hands with soap and warm water, and rinse off any fruits and vegetables required in the recipe. Make a healthy snack (**Banana Split Pudding Cups**) using foods that are high in nutrients. Talk to students about other foods that are healthy for their bodies and that they like to eat.

**Materials**

• cutting board

• knife (to slice fruit)

• paper towels

• plastic cups

• eating utensils

**Ingredients**

• chocolate and vanilla pudding

• bananas

• strawberries

• crushed pineapple

• granola

**Directions**

1. Peel bananas. Remove any green tops from strawberries. Use a cutting board and knife to slice strawberries and bananas.

2. Layer sliced bananas, chocolate pudding, strawberries, vanilla pudding, crushed pineapple, and granola. Make sure everyone shares.

3. Enjoy your treat!

**Last 10 minutes** – Clean up and answer final questions
